# Extracellular cap domain is an essential component of the TRPV1 gating mechanism

**DOI:** 10.1038/s41467-021-22507-3

**Published:** 2021-04-12

**Authors:** Kirill D. Nadezhdin, Arthur Neuberger, Yury A. Nikolaev, Lyle A. Murphy, Elena O. Gracheva, Sviatoslav N. Bagriantsev, Alexander I. Sobolevsky

**Affiliations:** 1 Department of Biochemistry and Molecular Biophysics, Columbia University, New York, NY USA; 2 Department of Cellular and Molecular Physiology, Yale University School of Medicine, New Haven, CT USA; 3 Department of Neuroscience, Yale University School of Medicine, New Haven, CT USA; 4 Program in Cellular Neuroscience, Neurodegeneration and Repair, Yale University School of Medicine, New Haven, CT USA

**Keywords:** Transient receptor potential channels, Electrophysiology, Permeation and transport, Cryoelectron microscopy

## Abstract

Transient receptor potential (TRP) channels are polymodal molecular sensors involved in numerous physiological processes and implicated in a variety of human diseases. Several structures of the founding member of the TRP channel family, TRPV1, are available, all of which were determined for the protein missing the N- and C-termini and the extracellular S5-P-loop. Here, we present structures of the full-length thirteen-lined ground squirrel TRPV1 solved by cryo-EM. Our structures resolve the extracellular cap domain formed by the S5-P-loops and the C-terminus that wraps around the three-stranded β-sheet connecting elements of the TRPV1 intracellular skirt. The cap domain forms a dome above the pore’s extracellular entrance, with four portals leading to the ion conductance pathway. Deletion of the cap increases the TRPV1 average conductance, reduces the open probability and affects ion selectivity. Our data show that both the termini and the cap domain are critical determinants of TRPV1 function.

## Abstract (web summary)

Structural and functional characterization of the full-length TRPV1 channel from the thirteen-lined ground squirrel reveal the architecture of the extracellular cap domain and the intracellular C-terminus, and suggest a role of the cap domain in TRPV1 conductance and ion selectivity.

## Introduction

In mammals, the detection of high temperature is carried out by terminal endings of primary afferent neurons expressing thermosensitive ion channels of the transient receptor potential (TRP) family^1^. Several mammalian species have evolved the ability to comfortably withstand high environmental heat for a long period of time, which allowed them to inhabit otherwise prohibitively hot ecological niches. Among these are small diurnal rodents, including 13-lined ground squirrels (*Ictidomys tridecemlineatus*), who developed tolerance to environmental heat via modification of the primary noxious heat sensor, TRPV1 (refs. ^2–4^). In contrast to TRPV1 orthologues from rat (rTRPV1) and other mammals, ground squirrel TRPV1 (sqTRPV1) fails to activate by heating in a physiologically relevant temperature range, even though the channel is sensitive to chemical agonists, such as acid and capsaicin^5^. The available structures of rTRPV1 revealed the overall architecture and key elements of the gating machinery^6–8^, but all of these structures were solved for a truncated construct that lacked the N- and C-termini, as well as the extracellular S5-P-loop. In fact, these missing regions have been implicated in the mechanism of temperature sensitivity^9–18^, which remains poorly understood.

In this work, we study the full-length sqTRPV1 using cryo-electron microscopy (cryo-EM) combined with electrophysiology and mutagenesis to understand the general mechanisms of TRPV1 function. We show that the S5-P-loops form an extracellular cap domain that shapes portals for ions to enter the central channel pore and is critical for channel conductance, open probability and ion selectivity. We also show that the C-terminus of the full-length TRPV1 wraps around the three-stranded β-sheet making numerous molecular interactions that strengthen intersubunit interfaces and stabilize the intracellular skirt.

## Results and discussion

### Apo-state structure

**Fig. 1:**
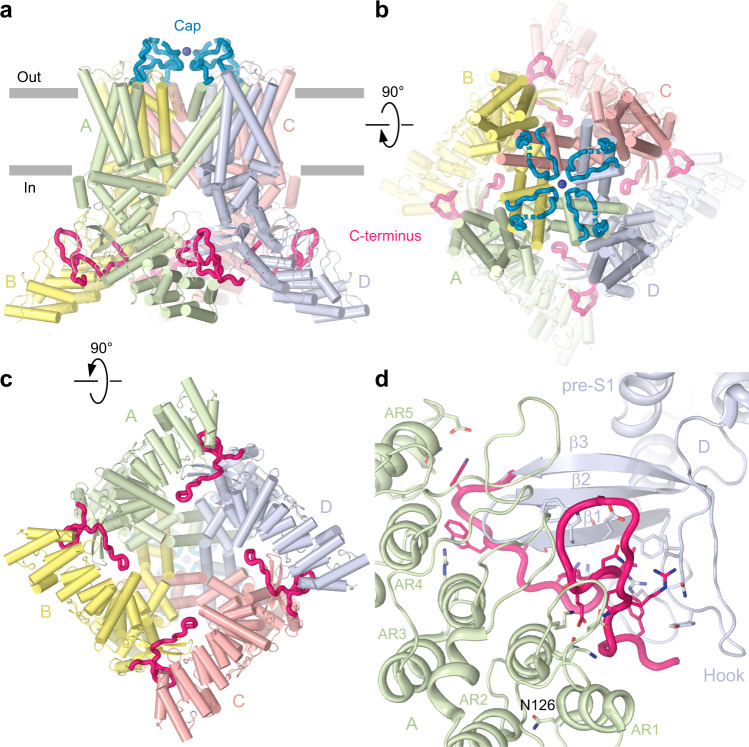
Structure of full-length sqTRPV1. **a**–**c** Side (**a**), top (**b**) and bottom (**c**) views of full-length sqTRPV1 structure with four subunits coloured differently, the cap domain in blue and the C-terminus in pink. Dark blue sphere is a putative chloride ion. **d** Close-up view of the C-terminus with the residues contributing to interactions with other domains shown in sticks. Asparagine N126, the serine substitution of which converts weakly temperature-sensitive sqTRPV1 into highly temperature-sensitive rTRPV1-like channel, is labelled.

We expressed full-length wild-type sqTRPV1 (ref. ^5^) in HEK 293 cells and subjected the purified apo protein to cryo-EM (Supplementary Fig. 1 and Supplementary Table 1). The resulting structure (Fig. 1a–c) has a similar overall shape and architecture, as the previously published structures of truncated rTRPV1 (refs. ^6–8^; Supplementary Fig. 2). However, of the three regions missing in rTRPV1—the N-terminus, the S5-P-loop and the C-terminus—the latter two appear to fold into structural elements in the context of full-length sqTRPV1. The C-terminus of sqTRPV1 represents an extended polypeptide that wraps around the three-stranded β-sheet (Fig. 1d), which connects the ankyrin repeat domains of the neighbouring subunits to form the typical for TRPV channels intracellular skirt. Electrostatic and hydrophobic interactions between the C-terminus, the three-stranded β-sheet and the C-terminal hook of one subunit, and the loops of ankyrin repeats AR2–5 of the neighbouring subunit make the intersubunit interfaces stronger. As a result, the intracellular skirt of full-length sqTRPV1 is less dynamic than that of truncated rTRPV1. Indeed, the entire skirt is well-resolved in the cryo-EM density of full-length sqTRPV1, while density for two peripheral of the six ankyrin repeats, as well as the C-terminal hook domain preceding the third strand of the three-stranded β-sheet in truncated rTRPV1 was missing^6–8^. Other than these differences, apo-state structures of sqTRPV1 and rTRPV1 are quite similar (Supplementary Fig. 2a, d), suggesting that whenever the complete termini are present in rTRPV1, they are likely to interact with the rest of the protein in the same way as in sqTRPV1.

Similar to sqTRPV1, the extended C-terminus that wraps around the three-stranded β-sheet has also been observed in cryo-EM structures of other temperature-sensitive TRPV channels TRPV2–3 (refs. ^19–22^). Compared to TRPV2–3, the C-terminus in the full-length sqTRPV1, after wrapping around the β-sheet, makes a U-turn and follows the edge of the skirt, providing additional interdomain interactions (Fig. 1d). Unwrapping of the C-terminus and binding of an otherwise unfolded region of the N-terminus have been recently shown to accompany temperature-induced opening of TRPV3 (ref. ^23^). Similarly, both N- and C-termini may contribute to temperature gating of TRPV1. Supporting this idea, the serine substitution of asparagine N126 converted weakly temperature-sensitive sqTRPV1 into highly temperature-sensitive rTRPV1-like channel^5^. N126 is located at the end of the AR1 helix that is in direct contact with AR2 helix contacting the C-terminus (Fig. 1d). Thus, alteration of the AR1–AR2 interaction is expected to have an immediate allosteric effect on the binding of the C-terminus. In contrast, in temperature-insensitive TRPV5–6, the C-terminus does not wrap around the three-stranded β-sheet, and the N-terminal helix, which pillars the corners of the intracellular skirt^24,25^, plays the role of an intersubunit “glue”.

### Extracellular cap

The S5-P-loops of the full-length sqTRPV1 assemble into an extracellular cap domain that forms a dome above the pore extracellular entry (Fig. 1a, b). The cryo-EM density for the cap is relatively weak and is completely missing for residues 608–617. This can be explained by the dynamic nature of the S5-P-loops and by possible spontaneous crosslinking of cysteines C623, which in the fourfold symmetrical model of sqTRPV1 come close together at the centre of the cap with 5.5 Å distances between their Cα atoms. In case such crosslinking does happen, the population of channels that contribute to cryo-EM reconstruction is likely heterogeneous and includes particles with non-symmetrical (one cysteine crosslink), twofold symmetrical (two crosslinks) and fourfold symmetrical (no crosslinks) cap domains.

**Fig. 2:**
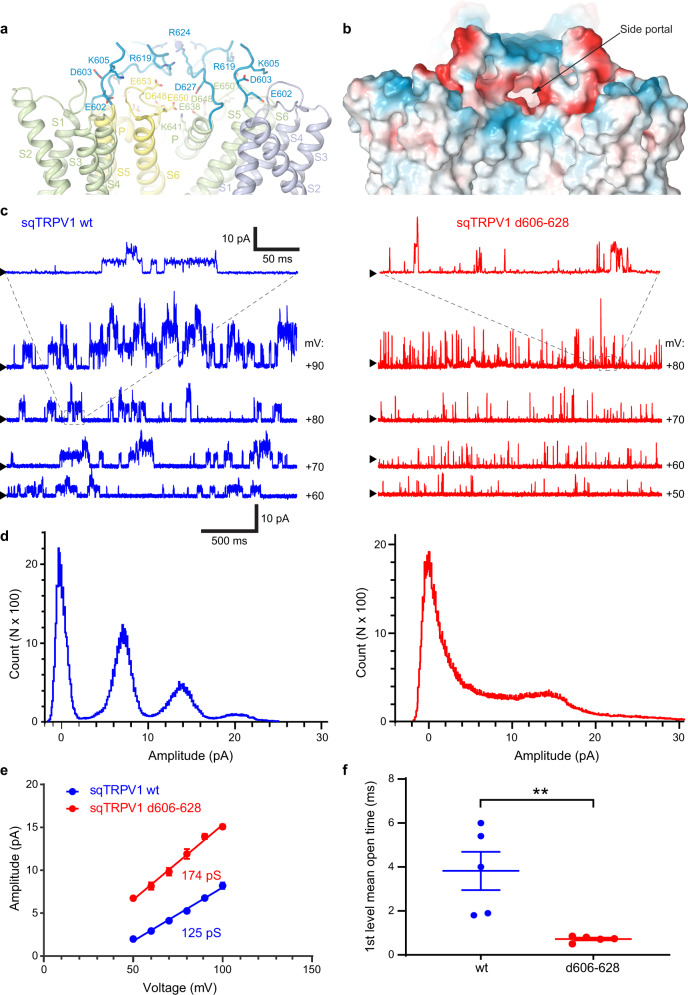
Cap domain and its role in TRPV1 function. **a** Ribbon diagram of the sqTRPV1 extracellular portion with subunits in different colour and the cap domain in blue. Charged residues are shown as sticks. **b** Surface view of the same region coloured by electrostatic potential with positively charged areas in blue, negatively charged in red and neutral in white. **c** Representative traces from single-channel recording of spontaneous openings of wild-type sqTRPV1 and d606–628 mutant at different voltages in cell-attached patch from HEK293T cells at room temperature. Black arrows indicate the position of the baseline current. Upward single-channel deflections represent outward current. **d** Amplitude histograms corresponding to the channel activity at 90 mV for wild-type sqTRPV1 and d606–628 mutant. **e** Current–voltage relationship for wild-type sqTRPV1 and d606–628 mutant with its single-channel conductances, fitted to the linear equation (wt, *N* = 5 cells; d606–628, *N* = 9 cells). Source data are provided as a Source data file. **f** Mean open dwell time of wild-type sqTRPV1 and d606–628 mutant channels (at 70 mV). Each dot represents individual cell (*N* = 5 cells for each construct). Data are mean ± SEM. **P = 0.0074, two-sided *t* test).

Strong density in the middle of the cap, which we interpreted as a chloride ion coordinated by arginines R624, represents a likely barrier for cation conductance along the central axis of the channel. On the other hand, four side portals in the dome lead to the ion-conducting pore extracellular entrance (Fig. 2a, b). Each portal is formed by the N-terminal part of the S5-P-loop of one subunit and the C-terminal part of the S5-P-loop of the neighbouring subunit, and contains numerous negatively charged residues. Correspondingly, the overall negative charge of each individual portal (Fig. 2b) creates a favourable environment for passage of cations, and suggests a possible role of the cap domain in TRPV1 conductance and ion selectivity. The missing residues 608–617 can potentially form a loop capable of occluding the side portals, thus providing an additional way to regulate the ion channel conductance.

To test the importance of the cap domain for the TRPV1 function, we generated a mutant channel lacking residues 606–628 (sqTRPV1 d606–628, Supplementary Fig. 3). Electrophysiological recordings in *Xenopus* oocytes showed that sqTRPV1 d606–628 lacks heat activation (Supplementary Fig. 4a), similar to wild-type channel^5^. The amplitude histograms of cell-attached single-channel recordings from HEK293T cells revealed that deletion of the cap domain produced a significant increase in single-channel conductance from 125 ± 4.9 pS for wild type to 174.6 ± 6.6 pS for the d606–628 mutant (*P* = 0.003, *t* test, Fig. 2c–e)^26^. Compared to wild-type sqTRPV1, the d606–628 mutant exhibited flickering behaviour, in part due to the significantly decreased mean open dwell time (Fig. 2f and Supplementary Fig. 4b). In addition, we expressed sqTRPV1 wt and sqTRPV1 d606–628 in *Xenopus* oocytes, and compared their reversal potential, *E*_rev_, by recording currents in response to different concentrations of capsaicin. At both 200 nM and 2 µM of capsaicin, sqTRPV1 wt and sqTRPV1 d606–628 showed significantly different values of *E*_rev_, suggesting that the deletion of the S5-P-loop affects ion selectivity of sqTRPV1 (Supplementary Fig. 4c). Together, our functional data show that the cap domain is an essential component of the TRPV1 gating mechanism.

### Lipids and ligands

**Fig. 3:**
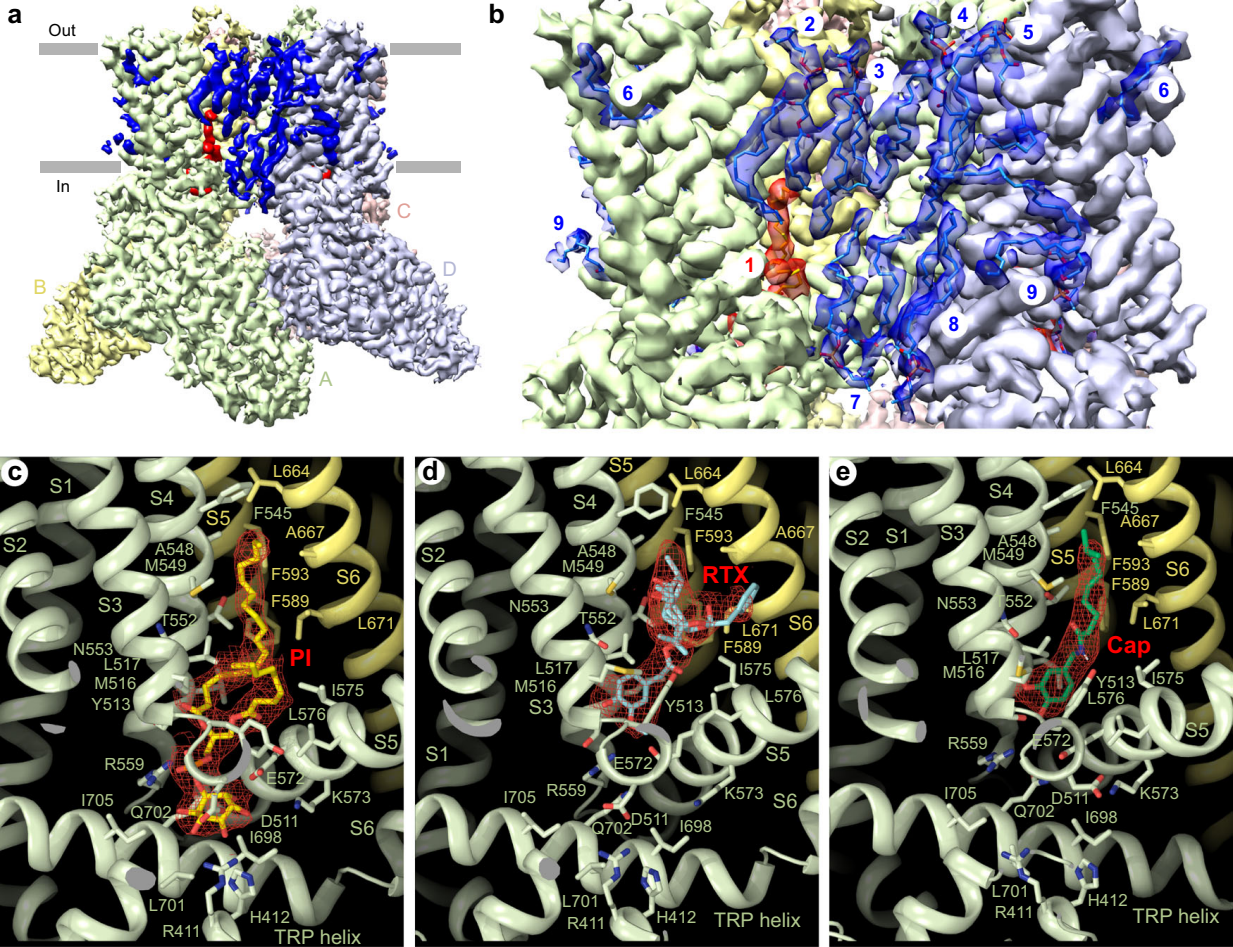
Lipids and ligands. **a** Cryo-EM density for the apo-state full-length sqTRPV1, with the lipid densities coloured red or blue. **b** Close-up view of the membrane region, with the molecules of lipid shown in sticks and numbered. **c**–**e** Close-up view of the vanilloid site (site 1 in **b**) in the apo-state structure harbouring the molecule of phosphatidylinositol (**c**), RTX-bound (**d**) and capsaicin-bound (**e**) structures. The molecules of phosphatidylinositol, RTX and capsaicin are shown as yellow, blue and green sticks, respectively, with the corresponding cryo-EM density shown in red.

The apo-state structure of the full-length sqTRPV1 shows at least nine well-resolved densities of annular lipids per channel subunit (Fig. 3a, b). The majority of densities have a clear head-and-two-tails appearance and were modelled with phosphatidylcholine. The exception is the lipid at site 1 or the vanilloid binding site, which in structures of rTRPV1 accommodates agonists resiniferatoxin (RTX) and capsaicin, and antagonist capsazepine^6–8^. Similar to the apo-state structure of rTRPV1 (ref. ^7^), the vanilloid site in the apo-state structure of sqTRPV1 contains density that is best fit by phosphatidylinositol (PI, Fig. 3c). The head of PI is accommodated by polar and charged residues of the linker domain (R411 and H412), the S2–S3 loop (D511 and Y513), the S4–S5 linker (R559, E572 and K573) and the TRP helix (Q702), while the PI tails face hydrophobic residues of S3 and S4 from one subunit, and S5 and S6 from the neighbouring subunit.

To get an insight into ligand binding to full-length TRPV1, we determined structures of sqTRPV1 in complex with agonists RTX and capsaicin, which both potently activate sqTRPV1, similar to the rat orthologue (Supplementary Fig. 4d)^5,27^. In agreement with the previous structures of rTRPV1, both RTX and capsaicin bind to the vanilloid site and are coordinated by residues in S2–S3 loop, S3, S4 and S4–S5 linker of one subunit, and S5 and S6 of the neighbouring subunit (Fig. 3d, e and Supplementary Fig. 5). In the apo state, the same residues contribute to the binding of PI, which resides deeper in the vanilloid site towards the linker domain and pushes away the side chain of tyrosine Y513. Binding of both RTX and capsaicin results in shortening the distance between the hydrogen bond-forming sidechains of T552 and E572, and movement of the S4–S5 linker away from the pore (Supplementary Fig. 5b, c). This S4–S5 linker movement was proposed to be coupled to S6 movement and pore opening^28^. While binding of PI, RTX and capsaicin to full-length sqTRPV1 is nearly identical to their binding to truncated rTRPV1, there is a notable difference in the resulting architecture of the ion channel pore.

### Ion channel pore

**Fig. 4:**
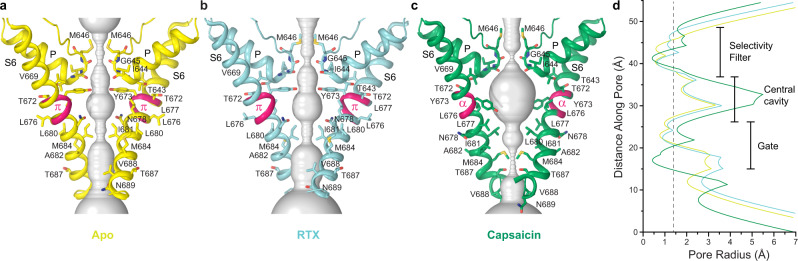
Ion channel pore. **a**–**c** Pore-forming domains in the apo (**a**), RTX-bound (**b**) and capsaicin-bound (**c**) states with the residues lining the pore shown as sticks. Only two of four subunits are shown, with the front and back subunits omitted for clarity. The pore profiles are shown as space-filling models (gray). The region that undergoes α-to-π transition in S6 is highlighted in pink. **d** The pore radius for the sqTRPV1 structures calculated using HOLE^47^. The vertical dashed line denotes the radius of a water molecule, 1.4 Å.

The pore of the full-length sqTRPV1 in the apo and RTX-bound states (Fig. 4a, b) resembles the pore of the truncated rTRPV1 in the apo state^6–8^ (Supplementary Fig. 2). Measurements of the pore radius confirm that both the selectivity filter and the lower gate of the channel are completely closed for permeation of water and ions (Fig. 4d). Interestingly, the presence of DKTX bound to rTRPV1 together with RTX leads to significant pore widening and opening of the channel^6–8^ (Supplementary Fig. 2e). It remains to be determined whether the open-state structure of rTRPV1 can be determined in the absence of DKTX. However, binding of RTX alone was not enough to capture the open-state structure of sqTRPV1, which can be opened functionally without help of DKTX (Supplementary Fig. 4d).

The narrow constriction at the lower gate of sqTRPV1 in the apo and RTX-bound states is formed by the S6 residue I681, which is homologous to I679 that determines the narrowest diameter of the lower pore in all published structures of rTRPV1 (refs. ^6–8^). In contrast, the narrow constriction of the lower pore in the capsaicin-bound structure of sqTRPV1 is formed by the S6 residue M684, while I681 faces away from the pore (Fig. 4c). This change occurs because S6, which contains a central π-bulge in other conformations of sqTRPV1 and rTRPV1, becomes entirely α-helical in the capsaicin-bound structure of sqTRPV1. While the α-helical S6 has not been observed in TRPV1 structures before^6–8^, it has been predicted^29^ and has also been observed in structures of other representatives of TRPV channels^21,22,24,30–32^. In fact, the α-to-π transition in S6, which results in ~100° rotation of the bottom portion of S6 and exposure of a completely different set of residues to the channel pore, has been shown experimentally by determining structures of TRPV6 (ref. ^33^), TRPV3 (refs. ^21,22^) and TRPV2 (ref. ^34^) in different conformations. By solving structures of sqTRPV1 in the apo, RTX-bound and capsaicin-bound states, we now demonstrate experimentally that the α-to-π transition in S6 can happen in TRPV1 and propose that this transition is an essential feature of TRPV1 gating.

Measurements of the pore radius suggest that the ion channel of sqTRPV1 in the capsaicin-bound state is in a closed non-conducting conformation (Fig. 4d). The channel is too narrow to conduct water or ions at both the selectivity filter and the lower pore, where the narrow constriction is formed by M684. In contrast, despite remaining non-conducting, the lower pore in the capsaicin-bound structure of truncated rTRPV1 is dilated compared to its apo-state structure^6–8^ (Supplementary Fig. 2f). Whether the difference in the pore size and the presence of the α-to-π transition in S6 originates from usage of different TRPV1 orthologs or from truncated versus full-length constructs of the channel remains to be determined. The fact that the pore of sqTRPV1 or rTRPV1 (refs. ^6–8^) remains closed, while the agonists RTX or capsaicin are bound to the channel suggests that the channel resides in inactivated states. Future studies are required to better understand the role of these conformations in the gating mechanism of TRPV1.

## Methods

### Construct

The full-length 13-lined ground squirrel TRPV1 (GenBank KU877439.1, residues 1–840)^5^ used in cryo-EM studies was cloned into a pEG BacMam vector^35^, followed by thrombin cleavage site (LVPRG), enhanced green fluorescent protein (eGFP) for monitoring during expression and a C-terminal streptavidin affinity tag (WSHPQFEK) for purification.

### Expression and purification

sqTRPV1 construct was expressed and purified as previously described for mTRPV3 (refs. ^22,23^), with minor modifications. Bacmids and baculoviruses were produced using a standard method^35^. Briefly, baculovirus was made in Sf9 cells for ~72 h (Thermo Fisher Scientific, mycoplasma test negative, GIBCO #12659017) and was added to the suspension adapted HEK 293 cells lacking N-acetyl-glucosaminyltransferase I (GnTI^−^, mycoplasma test negative, ATCC #CRL-3022) that were maintained in Freestyle 293 media (Gibco-Life Technologies #12338-018) supplemented with 2% FBS at 37 °C and 5% CO_2_. Twenty-four hours after transduction, 10 mM sodium butyrate was added to enhance protein expression, and the temperature was reduced to 30 °C. Seventy-two hours after transduction, the cells were harvested by centrifugation at 5471 × *g* for 15 min using a Sorvall Evolution RC centrifuge (Thermo Fisher Scientific), washed in phosphate-buffered saline (pH 8.0), and pelleted by centrifugation at 3202 × *g* for 10 min using an Eppendorf 5810 centrifuge. The cell pellet was resuspended in ice-cold lysis buffer, containing 20 mM Tris (pH 8.0), 150 mM NaCl, 0.8 μM aprotinin, 4.3 μM leupeptin, 2 μM pepstatin A, 1 mM phenylmethylsulfonyl fluoride and 1 mM β-mercaptoethanol (βME). Cells were subsequently lysed using a Misonix Sonicator with a preset programme (six cycles of 15 s “on” at the amplitude of 8 followed by 15 s “off”; this programme was repeated three times for optimal cell lysis) under constant stirring on ice. Unbroken cells and cell debris were pelleted using an Eppendorf 5810 centrifuge at 3202 × *g* and 4 °C for 10 min. The supernatant was subjected to ultracentrifugation in a Beckman Coulter ultracentrifuge using a Beckman Coulter Type 45Ti rotor at 186,000 × *g* and 4 °C for 1 h to pellet the membranes. The membrane pellet was mechanically homogenized and solubilized in the lysis buffer supplemented with 20 mM n-Dodecyl β-d-maltoside under stirring at 4 °C for 2 h. Insoluble material was removed by ultracentrifugation for 40 min in a Beckman Coulter Type 45Ti rotor at 186,000 × *g*, and the supernatant was added to strep resin and rotated for 14–16 h at 4 °C. Next, the resin was washed with ten column volumes of buffer containing 20 mM Tris (pH 8.0), 150 mM NaCl, 1 mM βME and 0.01% (w/v) glyco-diosgenin (GDN), and the protein was eluted with the same buffer supplemented with 2.5 mM d-desthiobiotin. The eluted protein was concentrated using a 100 kDa NMWL centrifugal filter (MilliporeSigma™ Amicon™) to 0.5 mg ml^−1^. The sample was then digested with thrombin (1:200 mass ratio of thrombin to eluted protein) for 1 h at 22 °C, and centrifuged in a Sorvall MTX 150 Micro-Ultracentrifuge (Thermo Fisher Scientific) using a S100AT4 rotor for 30 min at 66,000 × *g* and 4 °C before injecting into a size-exclusion chromatography (SEC) column. The protein was further purified using a Superose™ 6 10/300 GL SEC column attached to an AKTA FPLC (GE Healthcare) and equilibrated in 150 mM NaCl, 20 mM Tris, 1 mM βME and 0.01% GDN (pH 8.0). The tetrameric peak fractions were pooled and concentrated using 100 kDa NMWL centrifugal filter (MilliporeSigma™ Amicon™) to 2.4–3.8 mg ml^−1^.

### Cryo-EM sample preparation and data collection

Au/Au grids were prepared as described in the literature^36^. Briefly, grids were prepared by first coating C-flat (Protochips, Inc., Morrisville, NC) CF-1.2/1.3–2Au mesh holey carbon grids with ~60 nm gold, using an Edwards Auto 306 evaporator. Subsequently, an Ar/O_2_ plasma treatment (4 min, 50 watts, 35.0 sccm Ar, 11.5 sccm O_2_) was used to remove the carbon with a Gatan Solarus (model 950) Advanced Plasma Cleaning System (Gatan, Pleasanton, CA, USA). The grids were again plasma treated (H_2_/O_2_, 25 s, 10 watts, 6.4 sccm H_2_, 27.5 sccm O_2_) prior to sample application to make their surfaces hydrophilic.

RTX and capsaicin were dissolved in dimethyl sulfoxide (DMSO) to a concentration of 10 mM and 50 mM, respectively, and further diluted in buffer containing 20 mM Tris (pH 8.0), 150 mM NaCl, 1 mM βME and 0.01% GDN to a concentration of 1.5 mM. Ligands were added to protein 30 min before grid preparation at the final concentration of 50 μM (RTX) and 300 μM (capsaicin).

Images of frozen-hydrated particles of sqTRPV1 (3.8 mg ml^−1^) in apo state were collected on a Titan Krios transmission electron microscope (TEM; Thermo Fisher Scientific) operating at 300 kV, and equipped with a post-column GIF Quantum energy filter and a Gatan K3 Summit direct electron detection (DED) camera (Gatan, Pleasanton, CA, USA) using Leginon. A total number of 11,191 micrographs were collected in counting mode with an image pixel size of 1.06 Å and a defocus range of −0.8 to −2.5 µm, and energy filter slit of 30 eV. The total dose of ~65 e^−^ Å^−2^ was attained by using a dose rate of ~30 e^−^ pixel^−1^ s^−1^ across 50 frames for 2.5 s total exposure time.

Images for frozen-hydrated particles of sqTRPV1 (2.4 mg ml^−1^) in the presence of capsaicin were collected on a Titan Krios TEM (Thermo Fisher Scientific) operating at 300 kV, and equipped with a post-column GIF Quantum energy filter and a Gatan K3 Summit DED camera (Gatan, Pleasanton, CA, USA) using Leginon. A total number of 9453 micrographs were collected in counting mode with an image pixel size of 0.83 Å and a defocus range of −1.0 to −2.5 µm. The total dose of ~58.5 e^−^ Å^−2^ was attained by using a dose rate of ~16 e^−^ pixel^−1^ s^−1^ across 50 frames for 2.5 s total exposure time.

Images of frozen-hydrated particles of sqTRPV1 in the presence of RTX were collected on a Polara 300 kV TEM (FEI) with a Schottky field emission gun, cartridge loading system and a Gatan K3 Summit DED camera (Gatan, Pleasanton, CA, USA) using Leginon. A total number of 3253 micrographs were collected in counting mode with an image pixel size of 0.95 Å and a defocus range of −1.5 to −3.5 µm. The total dose of ~70.8 e^−^ Å^−2^ was attained by using a dose rate of ~16 e^−^ pixel^−1^ s^−1^ across 40 frames for 4 s of total exposure time.

### Image processing

All processing was completed in RELION^37^ and/or cryoSPARC^38^ (Supplementary Table 1). The initial drift and beam-induced motions were corrected using MotionCor2 (ref. ^39^) algorithm implemented in RELION, and contrast transfer function (CTF) estimation was performed using Gctf^40^. Following CTF estimation, micrographs were manually inspected and those with outliers in defocus values, ice thickness and astigmatism, as well as micrographs with lower predicted CTF-correlated resolution were excluded from the rest of the processing pipeline (individually assessed for each parameter relative to overall distribution; no set threshold). Initial set of particles was manually picked in RELION and further classified into 100 2D classes to create templates for the template-based picking in RELION that were used for the final round of picking. Picked particles were further 2D- and 3D-classified in iterative classification using RELION and/or cryoSPARC. The reported resolutions and local resolution predictions of the final maps were calculated in RELION and cryoSPARC, with the resolution range estimated by the gold standard Fourier shell correlation 0.143 criterion^41^. EM density visualization was done in UCSF Chimera^42^ and UCSF ChimeraX^43^.

### Model building

To build models of TRPV1 in Coot^44^, we used the previously published cryo-EM structures of TRPV1 as guides. The models were tested for overfitting by shifting their coordinates by 0.5 Å (using shake) in Phenix, refining each shaken model against a corresponding unfiltered half map, and generating densities from the resulting models in Chimera. Structures were visualized, and figures were prepared in UCSF Chimera, UCSF ChimeraX and Pymol^45^.

### Patch-clamp electrophysiology

sqTRPV1-pMO^5^ or sqTRPV1 d606–628-pMO constructs were co-transfected with pcDNA3-GFP into HEK293T^Δ*PIEZO1*^ (ref. ^46^) using Lipofectamine 3000 (Thermo Fisher), according to the manufacturer’s instructions. The following day, cells were plated onto glass coverslips coated with Matrigel Matrix (BD Bioscience) and analysed by voltage-clamp electrophysiology within 24 h after plating. Bath solution contained (in mM): 140 KCl, 10 HEPES, 1 MgCl_2_, 10 glucose, pH 7.3 (pH adjusted with KOH) and the pipette solution contained (in mM): 140 NaCl, 5 KCl, 10 HEPES, 1 EGTA, 1 MgCl_2_, pH 7.4 (pH adjusted with NaOH). To form the gigaohm seal, positive (+10 mmHg) pressure was maintained inside the electrode while approaching the cell, and released upon touching the cell membrane. Spontaneous single-channel events were recorded in the cell-attached mode at room temperature at indicated voltages. Single-channel openings are shown as upward deflections, representing outward current. Currents were sampled at 25 kHz and filtered at 1 kHz. Single-channel data was analysed using Clampfit 10.8. Mean single-channel amplitudes were calculated by fitting the current-amplitude histograms with Gaussian curves. The unitary conductance for sqTRPV1 was calculated by fitting the slope of the current–voltage relationship to the linear equation. NPo and mean open time values were calculated from 10 s recordings using Clampfit. RTX (Alomone, 2 mM stock in ethanol) was applied by perfusion.

### Oocyte electrophysiology

Defolliculated *Xenopus laevis* oocytes, stages V–VI (*Xenopus* 1), were injected with sqTRPV1 mRNA (10 ng) and assayed 48–72 h later by two-electrode voltage clamp in calcium-free ND96, containing (in mM): 3 KCl, 96 NaCl, 2 MgCl_2_ and 10 HEPES pH 7.4 (with NaOH), supplemented with 200 nM or 2 µM capsaicin. Currents were evoked by 200-ms voltage steps from −100 to 50 mV, in 10 mV increments, from a holding potential of 0 mV, and digitized at 2 kHz. The reversal potential was estimated from current–voltage plots. Capsaicin (Sigma-Aldrich, 1 mM stock in DMSO) was applied by perfusion. For temperature sensitivity experiments, currents were recorded in a gap-free mode at a holding potential of −80 mV. Temperature was controlled using the SC-20 in-line heater (Warner) and monitored by a thermistor placed in the chamber near the oocyte.

### Reporting summary

Further information on research design is available in the Nature Research Reporting Summary linked to this article.

## Supplementary information

Supplementary Information

Reporting Summary

## Data Availability

All data needed to evaluate the conclusions of the paper are present in the paper and/or the Supplementary Materials. Cryo-EM density maps have been deposited to the Electron Microscopy Data Bank (EMDB) under the accession codes EMD-23491 (sqTRPV1 apo), EMD-23492 (sqTRPV1 RTX), and EMD-23493 (sqTRPV1 capsaicin; see Supplementary Table 1). The corresponding model coordinates have been deposited to the PDB under accession numbers 7LQY (sqTRPV1 apo), 7LQZ (sqTRPV1 RTX), and 7LR0 (sqTRPV1 capsaicin; see Supplementary Table 1). All other data are available from the corresponding author upon request. Source data are provided with this paper.

## Source data

Source Data
