# Supplementary material for: Extracellular cap domain is an essential component of the TRPV1 gating mechanism
Source: Nat Commun. 2021 Apr 12;12:2154. doi: 10.1038/s41467-021-22507-3 (PMC8041747; doi:10.1038/s41467-021-22507-3)
Supplement: Supplementary file 1 — Supplementary Information [file 41467_2021_22507_MOESM1_ESM.pdf]

Supplementary Materials

Extracellular cap domain is an essential component of the TRPV1 gating mechanism

Kirill D. Nadezhdin[1]*, Arthur Neuberger[1]*, Yury A. Nikolaev[2], Lyle A. Murphy[2], Elena O. Gracheva[2,3,4], Sviatoslav N. Bagriantsev[2] and Alexander I. Sobolevsky[1#]

[1] Department of Biochemistry and Molecular Biophysics, Columbia University, New York, New York, USA

[2] Department of Cellular and Molecular Physiology, Yale University School of Medicine, New Haven, CT 06520, USA

[3] Department of Neuroscience, Yale University School of Medicine, New Haven, CT 06520, USA

[4] Program in Cellular Neuroscience, Neurodegeneration and Repair, Yale University School of Medicine, New Haven, CT 06520, USA

* These authors contributed equally to this work

[#] Correspondence to: as4005@cumc.columbia.edu

This PDF file includes:

Supplementary Figures 1-5

Supplementary Table 1

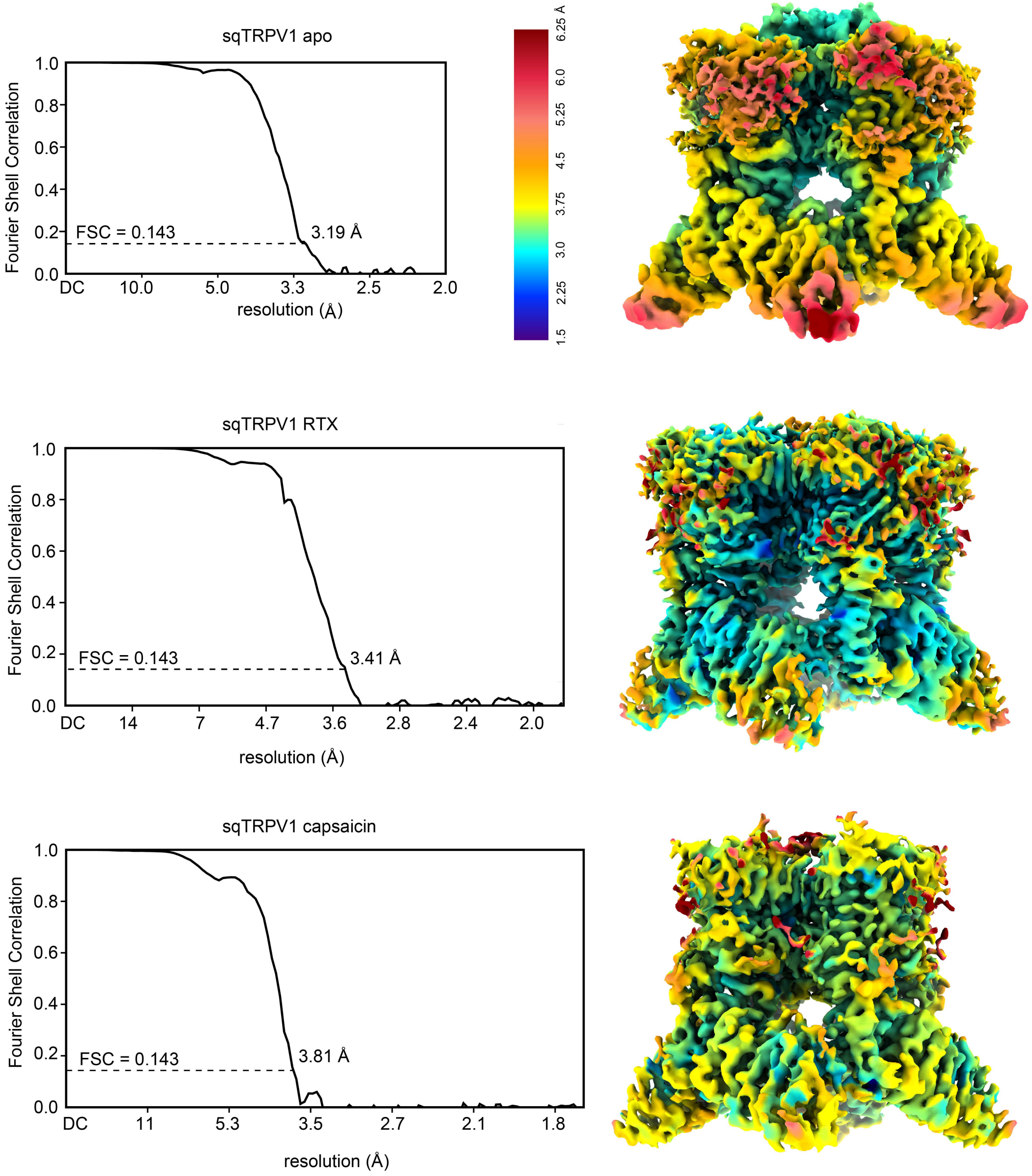

Supplementary Fig. 1. Characteristics of sqTRPV1 cryo-EM reconstructions. Plots show corrected FSC curves calculated between half maps, with the overall resolution estimated using the FSC = 0.143 criterion[41]. Cryo-EM maps are colored according to the local resolution estimation in Relion[37] or cryoSPARC[38].

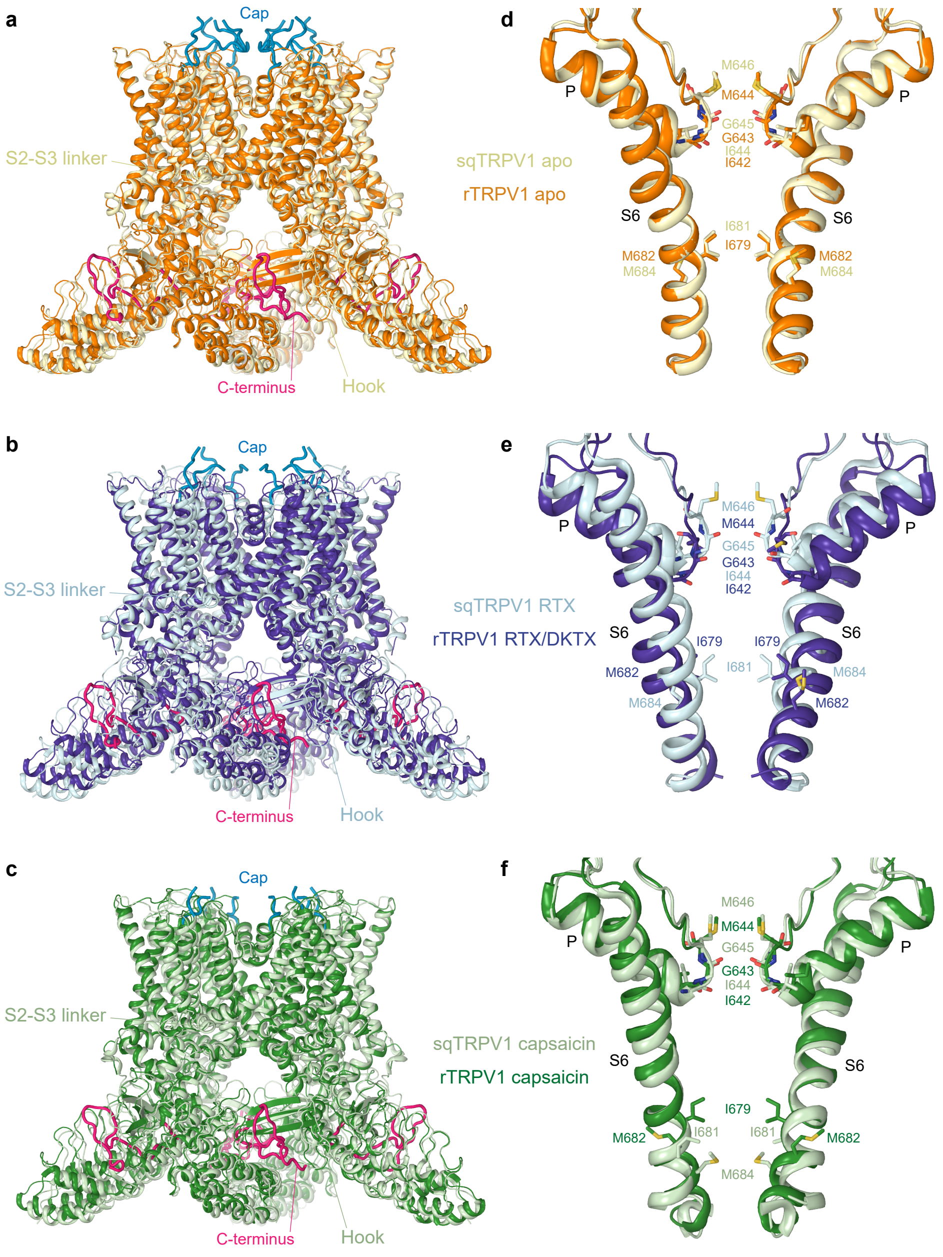

Supplementary Fig. 2. Comparison of squirrel and rat TRPV1 structures. Superposition of the entire structures viewed parallel to the membrane (**a-c**) and their pore-forming domains (**d-f**) in the apo state (**a,d**) colored yellow (sqTRPV1) and orange (rTRPV1, PDB ID: 3J5P), RTX- and RTX/DKTX-bound states (**b,e**) colored cyan (sqTRPV1) and blue (rTRPV1, PDB ID: 3J5Q), and capsaicin-bound state (**c,f**) colored light (sqTRPV1) and dark (rTRPV1, PDB ID: 3J5R) green. In (**a-c**), the unresolved in rTRPV1 structures cap domain (blue) and C-terminus (pink) as well as S2-S3 linker and C-terminal hook are labelled. In (**d-f**), only two of four subunits are shown, with the front and back subunits omitted for clarity, and residues that form the pore's narrow constrictions shown as sticks.

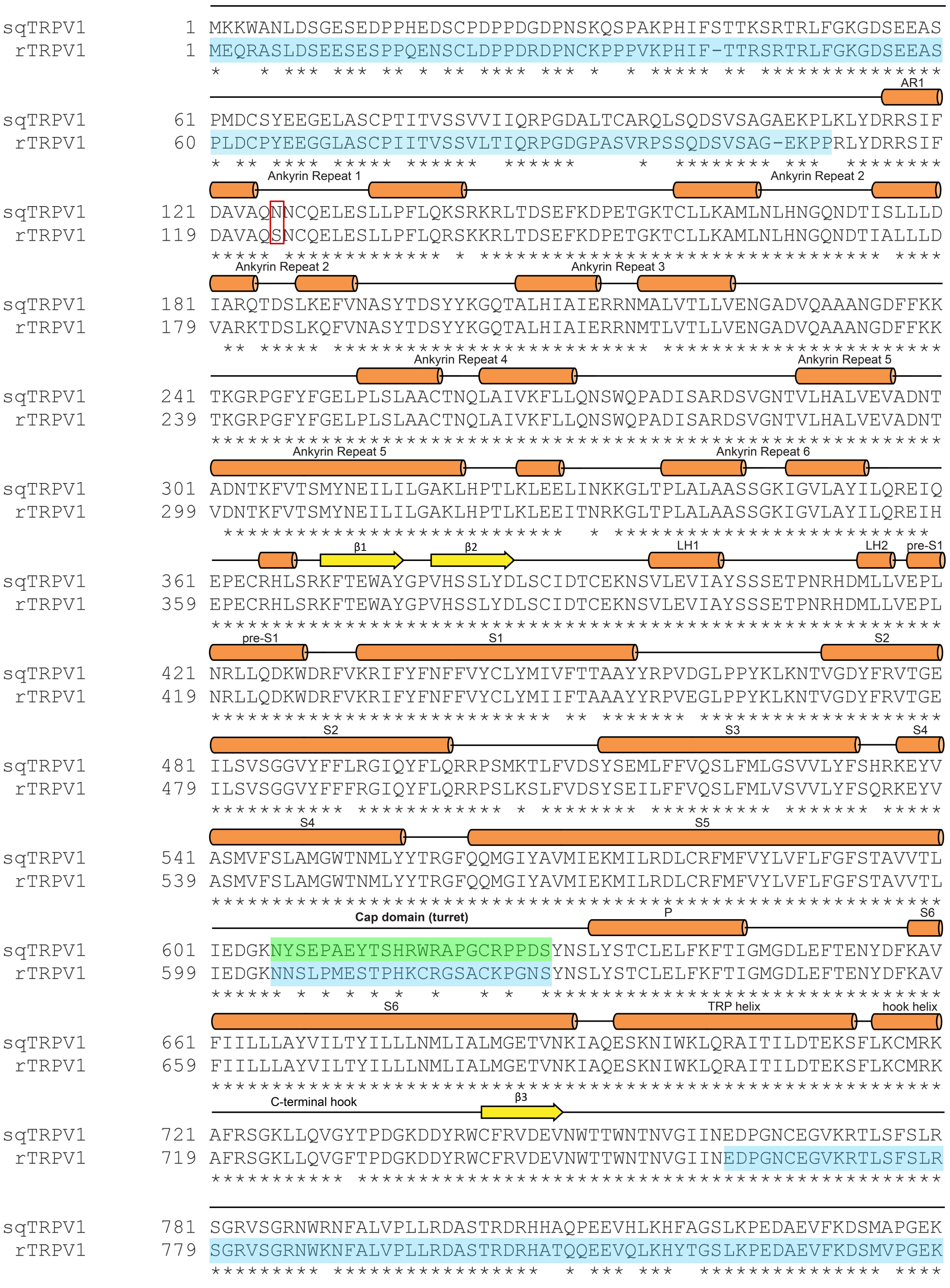

Supplementary Fig. 3. Sequence alignment of squirrel and rat TRPV1. α helices and β strands are depicted above the sequences as cylinders and arrows, respectively. The * symbols indicate residue conservation. Position of asparagine N126, the serine substitution of which converts weakly temperature-sensitive sqTRPV1 into highly temperature-sensitive rTRPV1-like channel, is indicated by the red rectangle. Regions deleted in structural constructs of rTRPV1[6-8] are highlighted in blue. The d606-628 deletion in sqTRPV1 is highlighted in green.

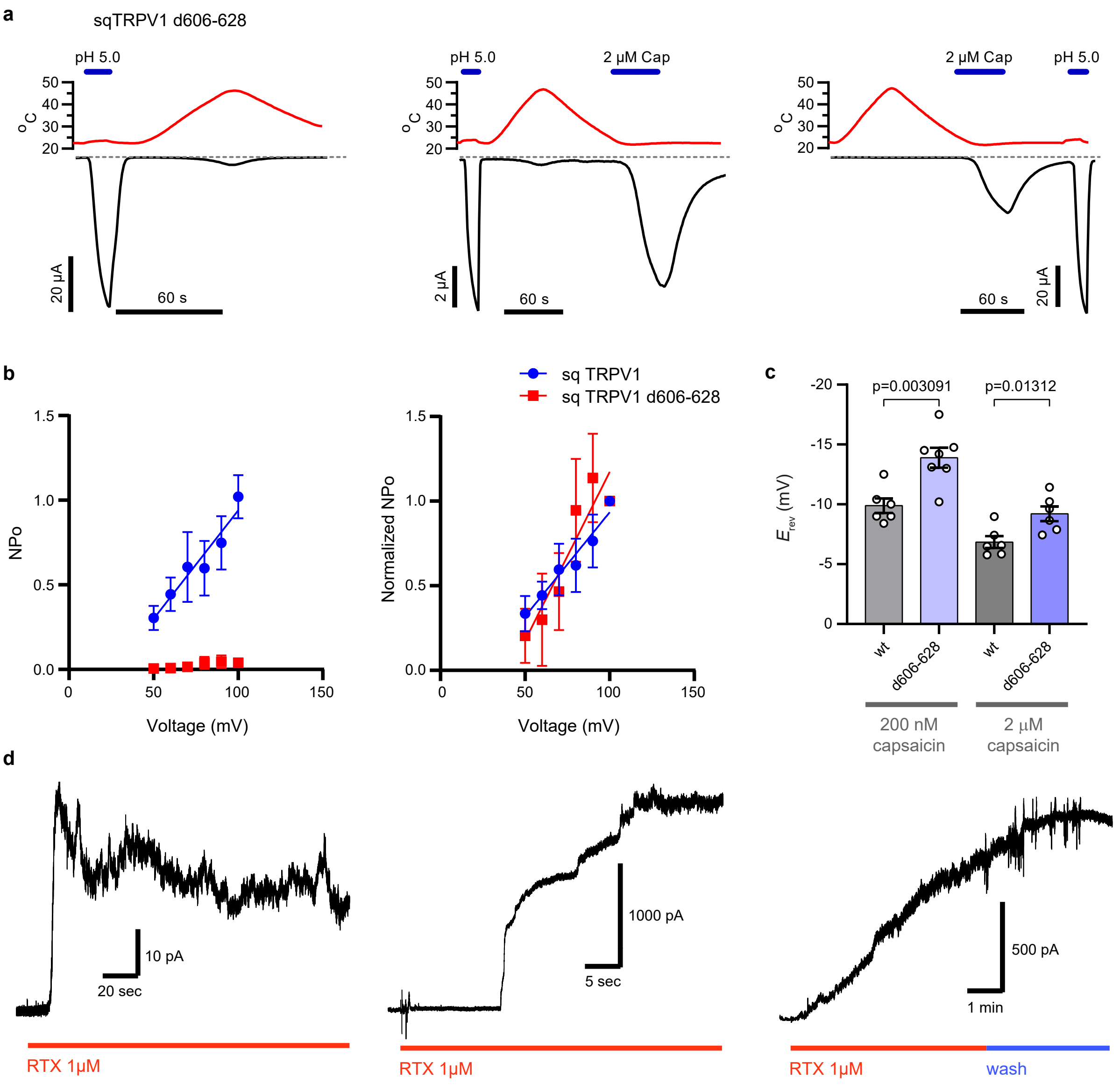

Supplementary Fig. 4. The deletion of the 606-628 region affects ion selectivity of sqTRPV1. (**a**) Squirrel TRPV1 d606-628 is not activated by heat. Shown are current traces recorded in *Xenopus* oocytes expressing sqTRPV1 d606-628 in response to acidic pH, capsaicin (Cap) or heat, using a gap-free protocol at a holding potential of -80mV. (**b**), Quantification of absolute (*left*) and 100 mV-normalized (*right*) relative open probability (NPo) for wild-type and d606-628 sqTRPV1 at different voltages, fitted to the linear equation (N = 5 for each construct). Data are mean ± SEM. Source data are provided as a Source Data file. (**c**) The reversal potential (E_{rev}) was measured in two oocytes batches by recording currents from *Xenopus* oocytes expressing sqTRPV1 wt or d606-628 mutant in response to application of capsaicin at 200 nM (wt, N = 6 cells; d606-628, N = 7 cells) or 2 µM (N = 6 cells for each construct). Significant difference in E_{rev} between sqTRPV1 wt and sqTRPV1 d606-628 suggests that the deletion of the S5-P-loop affects ion selectivity. Dots represent individual cells. Data are mean ± SEM. Statistics: unpaired two-sided t-test. Source data are provided as a Source Data file. **(d)** Current traces recorded using inside-out patch from sqTRPV1-expressing HEK293T cells at +80 mV, in the presence of 1 µM of RTX, representative of 7 cells.

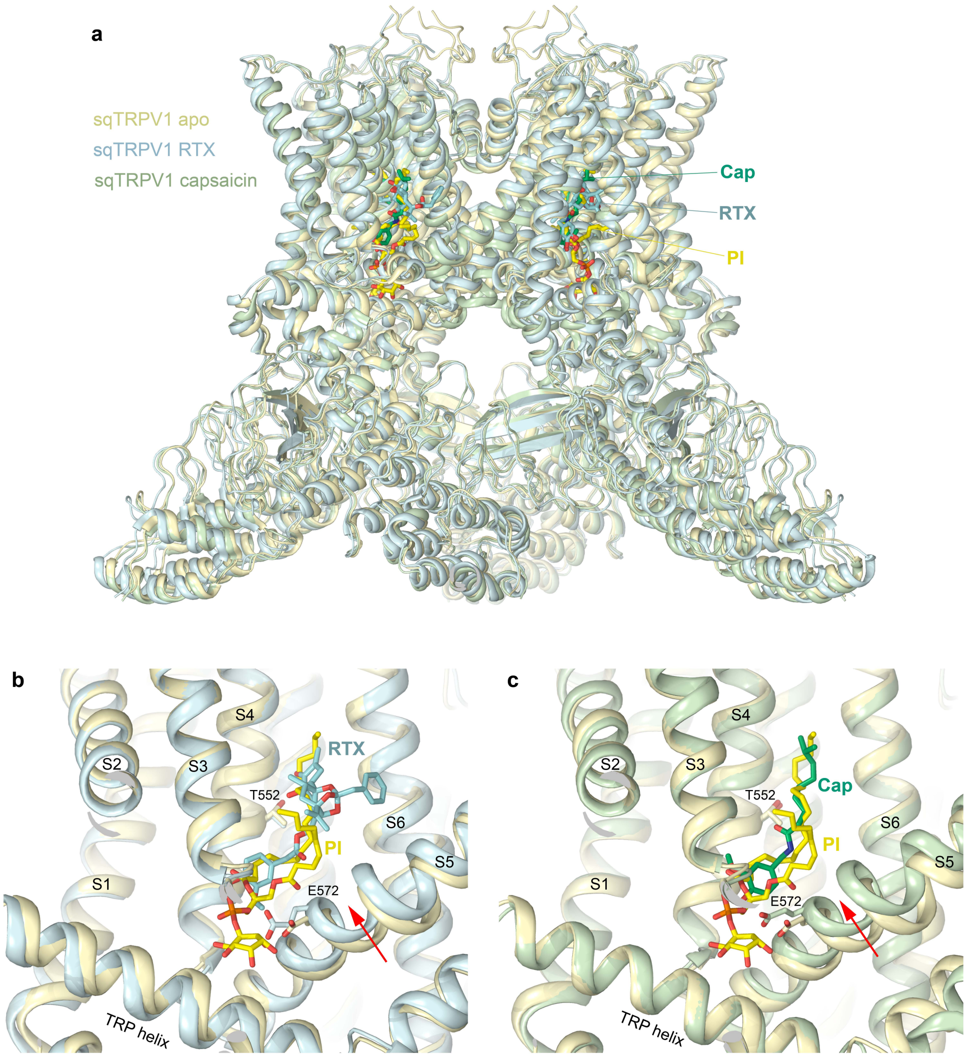

Supplementary Fig. 5. Comparison of the apo, RTX- and capsaicin-bound structures of sqTRPV1. a, Superposition of the apo (yellow), RTX-bound (blue) and capsaicin-bound (green) structures of sqTRPV1 viewed parallel to the membrane. **b-c**, Closeup view of the vanilloid site for superposition of the apo and RTX-bound structures (**b**) and apo and capsaicin-bound structures (**c**). The direction of the agonist-induced motion of the S4-S5 linker is indicated by red arrows. The molecules of phosphatidylinositol, RTX and capsaicin are shown as yellow, blue and green sticks, respectively.

Supplementary Table 1. Cryo-EM data collection, refinement and validation statistics.

Structure	sqTRPV1 apo	sqTRPV1 RTX	sqTRPV1 capsaicin
EMDB accession code	EMD-23491	EMD-23492	EMD-23493
PDB accession code	7LQY	7LQZ	7LR0
**Data collection and processing**			
Magnification	105,000	105,000	105,000
Voltage (kV)	300	300	300
Electron exposure ($e^{–}/Å^2$)	65	70.8	58.5
Defocus range (μm)	-0.8 to -2.5	-1.5 to -3.5	-1.0 to -2.5
Pixel size (Å)	1.06	0.95	0.83
Symmetry imposed	C4	C4	C4
Initial particle images (no.)	6,250,182	1,391,804	2,013,743
Final particle images (no.)	54,682	333,521	62,014
Map resolution (Å)	3.19	3.41	3.81
FSC threshold	0.143	0.143	0.143
**Refinement**			
Map sharpening *B* factor ($Å^2$)	-30	-153	-218
**Model composition**			
Non-hydrogen atoms	22,886	22,765	21,913
Protein residues	2,636	2,604	2,617
Ligands	34	37	33
**R.m.s. deviations**			
Bond lengths (Å)	0.006	0.009	0.010
Bond angles (°)	0.919	1.287	1.008
**Validation**			
Clashscore	4.69	6.50	2.99
Poor rotamers (%)	0.34	0.35	0.00
**Ramachandran plot**			
Favoured (%)	93.27	92.74	88.36
Allowed (%)	6.57	7.26	11.33
Disallowed (%)	0.15	0.00	0.31
